# Supplementary material for: Social buffering and contact transmission: network connections have beneficial and detrimental effects on Shigella infection risk among captive rhesus macaques
Source: PeerJ. 2016 Oct 27;4:e2630. doi: 10.7717/peerj.2630 (PMC5088628; doi:10.7717/peerj.2630)
Supplement: Figure S1 — In each plot, the Y-Axis represents the network metric plotted against network degree (a standard network metric) the X-axis. Black dots represent infected individuals (Group I: 23 of 100; Group II: 8 of 96; Group III: 7 of 99). Infection seems similarly concentrated among those with moderate-to-low degree and betweenness scores for Groups I and II, but more randomly scattered for Group III. [file peerj-04-2630-s001.pdf]

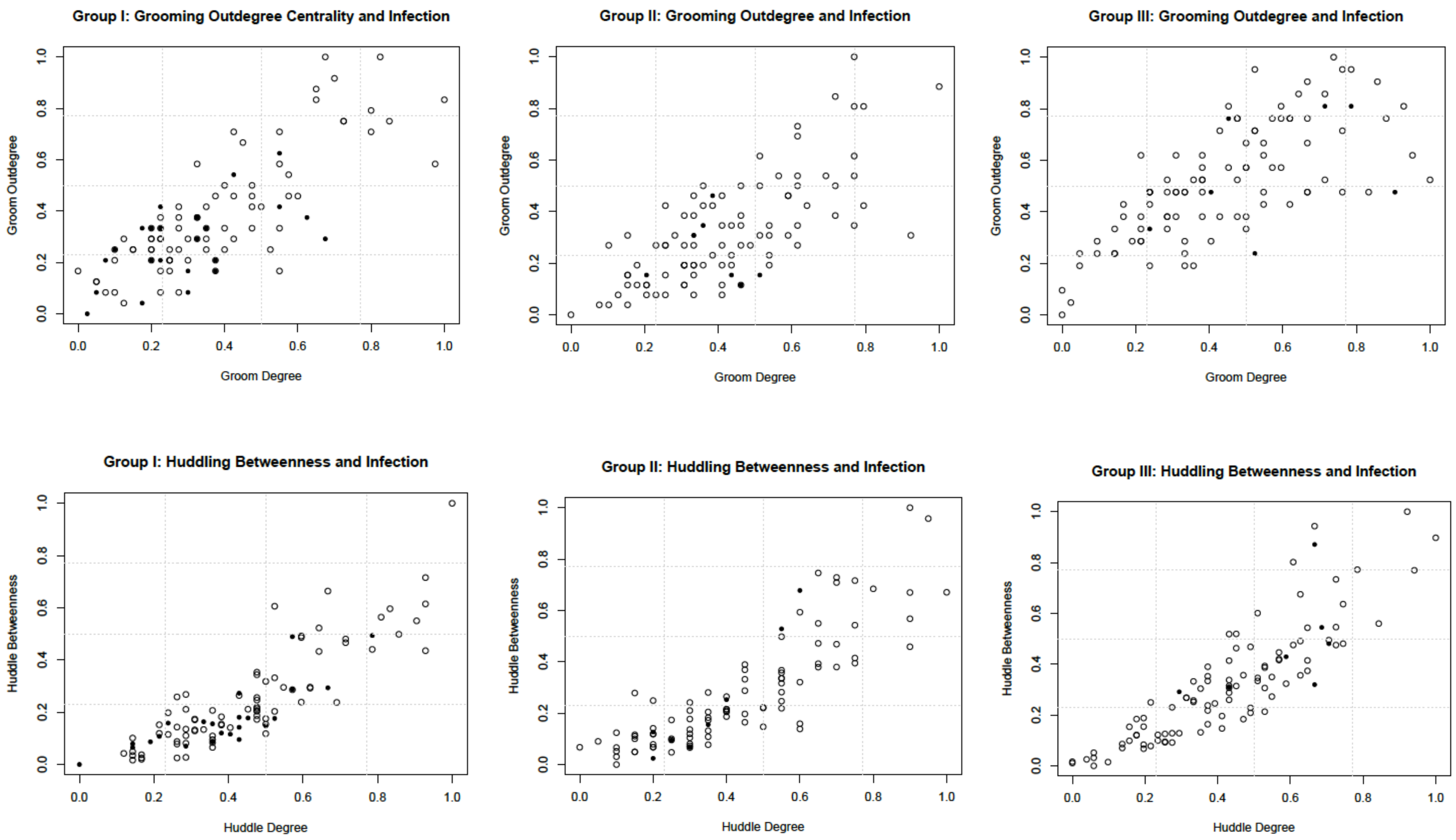

Group I: Grooming Outdegree Centrality and Infection
Groom Outdegree
Groom Degree
Group II: Grooming Outdegree and Infection
Groom Outdegree
Groom Degree
Group III: Grooming Outdegree and Infection
Groom Outdegree
Groom Degree
Group I: Huddling Betweenness and Infection
Huddle Betweenness
Huddle Degree
Group II: Huddling Betweenness and Infection
Huddle Betweenness
Huddle Degree
Group III: Huddling Betweenness and Infection
Huddle Betweenness
Huddle Degree
