# Supplementary material for: Social buffering and contact transmission: network connections have beneficial and detrimental effects on Shigella infection risk among captive rhesus macaques
Source: PeerJ. 2016 Oct 27;4:e2630. doi: 10.7717/peerj.2630 (PMC5088628; doi:10.7717/peerj.2630)
Supplement: Table S1 [file peerj-04-2630-s002.docx]

| Group ID | Number of Matrilines | Age (mean±SD) | Max. Age | Min. Age | Year of formation |
| --- | --- | --- | --- | --- | --- |
| I | 13 | 8.02±5.39 | 29 | 3 | 1991 |
| II | 13 | 8.30±4.69 | 21 | 3 | 1995 |
| III | 26 | 5.94±2.54 | 11 | 3 | 2005 |
