# Supplementary material for: Social buffering and contact transmission: network connections have beneficial and detrimental effects on Shigella infection risk among captive rhesus macaques
Source: PeerJ. 2016 Oct 27;4:e2630. doi: 10.7717/peerj.2630 (PMC5088628; doi:10.7717/peerj.2630)
Supplement: Table S2 — Parameters in ‘Bold’ font represent those selected as candidate model-sets from each dataset, based on Δ < 2 and parsimony-based selection criteria. [file peerj-04-2630-s003.docx]

| **Model Number** | **Model** | **Model Parameters** | | | | | **Summary Statistics** | | | |  |
| --- | --- | --- | --- | --- | --- | --- | --- | --- | --- | --- | --- |
|  |  | **AIC** | **AICc** | **Δ** | **w** | **df** | **Predictor(s)** | **Β** | **Adj SE** | **P** |  |
| 1 (Null) | Shigella ~ 1 | 173.50 | 173.52 | 4.09 | 0.02 | 196 |  |  |  |  |  |
| 2 | Shigella ~DR * Sex | 178.95 | 179.16 | 9.73 | 0 | 193 | DR | -0.56 | 0.90 | 0.54 |  |
|  |  |  |  |  |  |  | Sex | -0.31 | 0.76 | 0.68 |  |
|  |  |  |  |  |  |  | DR*Sex | 0.23 | 1.51 | 0.88 |  |
| **3** | **Shigella ~ GO** | **171.00** | **171.06** | **1.63** | **0.05** | **195** | **GO** | **-2.31** | **1.16** | **0.05*** |  |
| 4 | Shigella ~ GI | 175.11 | 175.17 | 5.74 | 0.01 | 195 | GI | -0.64 | 1.05 | 0.54 |  |
| 5 | Shigella ~ GB | 171.64 | 171.70 | 2.27 | 0.04 | 195 | GB | -2.23 | 1.25 | 0.08 |  |
| **6** | **Shigella ~ GE** | **169.37** | **169.43** | **0.00** | **0.12** | **195** | **GE** | **-2.76** | **1.22** | **0.02*** |  |
| 7 | Shigella ~ HD | 172.92 | 172.99 | 3.56 | 0.02 | 195 | HD | -1.50 | 0.96 | 0.12 |  |
| **8** | **Shigella ~ HB** | **171.20** | **171.26** | **1.84** | **0.05** | **195** | **HB** | **-2.38** | **1.27** | **0.06** |  |
| 9 | Shigella ~ HE | 173.91 | 173.97 | 4.54 | 0.01 | 195 | HE | -1.09 | 0.88 | 0.22 |  |
| 10 | Shigella ~ ADO | 175.22 | 175.28 | 5.86 | 0.01 | 195 | ADO | -0.44 | 0.84 | 0.60 |  |
| 11 | Shigella ~ ADI | 175.41 | 175.48 | 6.05 | 0.01 | 195 | ADI | 0.25 | 0.86 | 0.78 |  |
| 12 | Shigella ~ ASO | 175.39 | 175.45 | 6.02 | 0.01 | 195 | ASO | 0.32 | 0.97 | 0.74 |  |
| 13 | Shigella ~ ASI | 175.39 | 175.45 | 6.03 | 0.01 | 195 | ASI | 0.33 | 1.04 | 0.75 |  |
| 14 | Shigella ~ DC * GO | 171.29 | 171.50 | 2.07 | 0.04 | 193 | DC | 3.98 | 2.19 | 0.07 |  |
|  |  |  |  |  |  |  | GO | 2.18 | 2.51 | 0.38 |  |
|  |  |  |  |  |  |  | DC*GO | -13.02 | 6.88 | 0.06 |  |
| 15 | Shigella ~ DC * GI | 178.97 | 179.18 | 9.75 | 0.00 | 193 | DC | 0.09 | 1.74 | 0.96 |  |
|  |  |  |  |  |  |  | GI | -1.03 | 2.33 | 0.66 |  |
|  |  |  |  |  |  |  | DC*GI | 0.64 | 4.42 | 0.88 |  |
| 16 | Shigella ~ DC * GB | 175.34 | 175.55 | 6.12 | 0.01 | 193 | DC | 0.18 | 1.37 | 0.89 |  |
|  |  |  |  |  |  |  | GB | -2.94 | 2.91 | 0.31 |  |
|  |  |  |  |  |  |  | DC*GB | 1.42 | 5.90 | 0.81 |  |
| 17 | Shigella ~ DC * GE | 170.42 | 170.63 | 1.20 | 0.07 | 193 | DC | 2.99 | 1.95 | 0.13 |  |
|  |  |  |  |  |  |  | GE | 1.07 | 2.42 | 0.66 |  |
|  |  |  |  |  |  |  | DC*GE | -11.43 | 6.67 | 0.09 |  |
| 18 | Shigella ~ DC * HD | 176.40 | 176.61 | 7.18 | 0.00 | 193 | DC | -1.30 | 1.87 | 0.49 |  |
|  |  |  |  |  |  |  | HD | -2.78 | 2.07 | 0.18 |  |
|  |  |  |  |  |  |  | DC*HD | 3.21 | 4.61 | 0.49 |  |
| **19** | **Shigella ~ DC * HB** | **170.14** | **170.34** | **0.92** | **0.08** | **193** | **DC** | **-2.49** | **1.43** | **0.08** |  |
|  |  |  |  |  |  |  | **HB** | **-8.61** | **3.35** | **0.01*** |  |
|  |  |  |  |  |  |  | **DC*HB** | **13.55** | **6.07** | **0.03*** |  |
| 20 | Shigella ~ DC * HE | 177.78 | 177.99 | 8.56 | 0.00 | 193 | DC | -0.08 | 1.44 | 0.96 |  |
|  |  |  |  |  |  |  | HE | -0.93 | 1.68 | 0.58 |  |
|  |  |  |  |  |  |  | DC*HE | -0.74 | 4.06 | 0.86 |  |
| 21 | Shigella ~ GO + ADO | 172.93 | 173.06 | 3.63 | 0.02 | 194 | GO | -2.27 | 1.17 | 0.05 |  |
|  |  |  |  |  |  |  | ADO | -0.21 | 0.82 | 0.80 |  |
| 22 | Shigella ~ GO + ADI | 172.99 | 173.11 | 3.68 | 0.02 | 194 | GO | -2.30 | 1.17 | 0.05 |  |
|  |  |  |  |  |  |  | ADI | 0.10 | 0.84 | 0.90 |  |
| 23 | Shigella ~ GO + ASO | 172.77 | 172.9 | 3.47 | 0.02 | 194 | GO | -2.35 | 1.17 | 0.04 |  |
|  |  |  |  |  |  |  | ASO | 0.46 | 0.95 | 0.63 |  |
| 24 | Shigella ~ GO + ASI | 172.99 | 173.12 | 3.69 | 0.02 | 194 | GO | -2.30 | 1.17 | 0.05 |  |
|  |  |  |  |  |  |  | ASI | 0.09 | 1.02 | 0.93 |  |
| 25 | Shigella ~ GI + ADO | 177.03 | 177.16 | 7.73 | 0.00 | 194 | GI | -0.50 | 1.17 | 0.67 |  |
|  |  |  |  |  |  |  | ADO | -0.25 | 0.94 | 0.79 |  |
| 26 | Shigella ~ GI + ADI | 177.10 | 177.22 | 7.8 | 0.00 | 194 | GI | -0.62 | 1.11 | 0.58 |  |
|  |  |  |  |  |  |  | ADI | 0.07 | 0.92 | 0.94 |  |
| 27 | Shigella ~ GI + ASO | 176.70 | 176.82 | 7.39 | 0.00 | 194 | GI | -0.95 | 1.17 | 0.42 |  |
|  |  |  |  |  |  |  | ASO | 0.69 | 1.06 | 0.52 |  |
| 28 | Shigella ~ GI + ASI | 177.08 | 177.20 | 7.77 | 0.00 | 194 | GI | -0.60 | 1.08 | 0.58 |  |
|  |  |  |  |  |  |  | ASI | 0.18 | 1.08 | 0.86 |  |
| 29 | Shigella ~ GB + ADO | 173.63 | 173.76 | 4.33 | 0.01 | 194 | GB | -2.21 | 1.28 | 0.08 |  |
|  |  |  |  |  |  |  | ADO | -0.04 | 0.85 | 0.96 |  |
| 30 | Shigella ~ GB + ADI | 173.63 | 173.75 | 4.33 | 0.01 | 194 | GB | -2.24 | 1.27 | 0.08 |  |
|  |  |  |  |  |  |  | ADI | -0.06 | 0.86 | 0.95 |  |
| 31 | Shigella ~ GB + ASO | 173.15 | 173.27 | 3.85 | 0.02 | 194 | GB | -2.38 | 1.28 | 0.06 |  |
|  |  |  |  |  |  |  | ASO | 0.69 | 0.97 | 0.48 |  |
| 32 | Shigella ~ GB + ASI | 173.63 | 173.76 | 4.33 | 0.01 | 194 | GB | -2.24 | 1.27 | 0.08 |  |
|  |  |  |  |  |  |  | ASI | -0.06 | 1.05 | 0.95 |  |
| 33 | Shigella ~ GE + ADO | 171.28 | 171.41 | 1.98 | 0.04 | 194 | GE | -2.73 | 1.22 | 0.03 |  |
|  |  |  |  |  |  |  | ADO | -0.24 | 0.82 | 0.77 |  |
| 34 | Shigella ~ GE + ADI | 171.33 | 171.45 | 2.02 | 0.04 | 194 | GE | -2.76 | 1.22 | 0.02 |  |
|  |  |  |  |  |  |  | ADI | 0.16 | 0.83 | 0.84 |  |
| 35 | Shigella ~ GE + ASO | 171.17 | 171.29 | 1.87 | 0.05 | 194 | GE | -2.79 | 1.22 | 0.02 |  |
|  |  |  |  |  |  |  | ASO | 0.43 | 0.95 | 0.65 |  |
| 36 | Shigella ~ GE + ASI | 171.34 | 171.47 | 2.04 | 0.04 | 194 | GE | -2.75 | 1.22 | 0.02 |  |
|  |  |  |  |  |  |  | ASI | 0.16 | 1.01 | 0.88 |  |
| 37 | Shigella ~ HD + ADO | 174.69 | 174.82 | 5.39 | 0.01 | 194 | HD | -1.48 | 0.96 | 0.12 |  |
|  |  |  |  |  |  |  | ADO | -0.40 | 0.83 | 0.63 |  |
| 38 | Shigella ~ HD + ADI | 174.70 | 174.82 | 5.39 | 0.01 | 194 | HD | -1.55 | 0.97 | 0.11 |  |
|  |  |  |  |  |  |  | ADI | 0.41 | 0.86 | 0.63 |  |
| 39 | Shigella ~ HD + ASO | 174.83 | 174.95 | 5.52 | 0.01 | 194 | HD | -1.50 | 0.96 | 0.12 |  |
|  |  |  |  |  |  |  | ASO | 0.30 | 0.95 | 0.75 |  |
| 40 | Shigella ~ HD + ASI | 174.63 | 174.75 | 5.32 | 0.01 | 194 | HD | -1.58 | 0.98 | 0.11 |  |
|  |  |  |  |  |  |  | ASI | 0.57 | 1.04 | 0.58 |  |
| 41 | Shigella ~ HB + ADO | 173.13 | 173.25 | 3.82 | 0.02 | 194 | HB | -2.34 | 1.27 | 0.07 |  |
|  |  |  |  |  |  |  | ADO | -0.23 | 0.83 | 0.79 |  |
| 42 | Shigella ~ HB + ADI | 173.10 | 173.23 | 3.80 | 0.02 | 194 | HB | -2.39 | 1.27 | 0.06 |  |
|  |  |  |  |  |  |  | ADI | 0.27 | 0.85 | 0.75 |  |
| 43 | Shigella ~ HB + ASO | 173.00 | 173.13 | 3.70 | 0.02 | 194 | HB | -2.42 | 1.28 | 0.06 |  |
|  |  |  |  |  |  |  | ASO | 0.43 | 0.96 | 0.65 |  |
| 44 | Shigella ~ HB + ASI | 173.06 | 173.19 | 3.76 | 0.02 | 194 | HB | -2.40 | 1.27 | 0.06 |  |
|  |  |  |  |  |  |  | ASI | 0.39 | 1.03 | 0.71 |  |
| 45 | Shigella ~ HE + ADO | 175.48 | 175.60 | 6.17 | 0.01 | 194 | HE | -1.15 | 0.89 | 0.20 |  |
|  |  |  |  |  |  |  | ADO | -0.55 | 0.84 | 0.51 |  |
| 46 | Shigella ~ HE + ADI | 175.58 | 175.70 | 6.27 | 0.01 | 194 | HE | -1.20 | 0.91 | 0.19 |  |
|  |  |  |  |  |  |  | ADI | 0.50 | 0.87 | 0.57 |  |
| 47 | Shigella ~ HE + ASO | 175.86 | 175.99 | 6.56 | 0.00 | 194 | HE | -1.07 | 0.89 | 0.23 |  |
|  |  |  |  |  |  |  | ASO | 0.21 | 0.97 | 0.83 |  |
| 48 | Shigella ~ HE + ASI | 175.55 | 175.68 | 6.25 | 0.01 | 194 | HE | -1.20 | 0.91 | 0.19 |  |
|  |  |  |  |  |  |  | ASI | 0.63 | 1.05 | 0.55 |  |

DR: Dominance Rank; DC: Dominance Certainty; GO: Groom Outdegree; GI: Groom Indegree; GB: Groom Betweenness; GE: Groom Eigenvector; HD: Huddle Degree; HB: Huddle Betweenness; HE: Huddle Eigenvector; ADO: Aggression Outdegree; ADI: Aggression Indegree; ASO: Aggression Outstrength; ASI: Aggression Instrength

Δ: Difference in AICc from the best-fit model

W: Absolute model weight

*p < 0.05 **p < 0.01
