# Supplementary material for: Social buffering and contact transmission: network connections have beneficial and detrimental effects on Shigella infection risk among captive rhesus macaques
Source: PeerJ. 2016 Oct 27;4:e2630. doi: 10.7717/peerj.2630 (PMC5088628; doi:10.7717/peerj.2630)
Supplement: Table S3 — Parameters in ‘Bold’ font represent those selected as candidate model-sets from each dataset, based on Δ < 2 and parsimony-based selection criteria. [file peerj-04-2630-s004.docx]

| **Model Number** | **Model** | **Model Parameters** | | | | | **Summary Statistics** | | | |  |
| --- | --- | --- | --- | --- | --- | --- | --- | --- | --- | --- | --- |
|  |  | **AIC** | **AICc** | **Δ** | **w** | **df** | **Predictor(s)** | **Β** | **Adj SE** | **P** |  |
| 1 (Null) | Shigella ~ 1 | 52.73 | 52.77 | 7.25 | 0.00 | 99 |  |  |  |  |  |
| 2 | Shigella ~DR * Sex | 54.29 | 54.71 | 9.2 | 0.00 | 96 | DR | 3.54 | 2.36 | 0.13 |  |
|  |  |  |  |  |  |  | Sex | 0.30 | 2.75 | 0.91 |  |
|  |  |  |  |  |  |  | DR*Sex | -0.39 | 3.67 | 0.92 |  |
| 3 | Shigella ~ GO | 54.49 | 54.61 | 9.09 | 0.00 | 98 | GO | 0.87 | 1.77 | 0.62 |  |
| 4 | Shigella ~ GI | 52.13 | 52.25 | 6.73 | 0.01 | 98 | GI | 2.74 | 1.66 | 0.10 |  |
| 5 | Shigella ~ GB | 53.70 | 53.82 | 8.3 | 0.00 | 98 | GB | 2.01 | 1.85 | 0.28 |  |
| 6 | Shigella ~ GE | 52.54 | 52.66 | 7.15 | 0.00 | 98 | GE | 2.29 | 1.55 | 0.14 |  |
| 7 | Shigella ~ HD | 51.41 | 51.54 | 6.02 | 0.01 | 98 | HD | 3.31 | 1.87 | 0.08 |  |
| 8 | Shigella ~ HB | 52.19 | 52.31 | 6.79 | 0.01 | 98 | HB | 2.54 | 1.55 | 0.10 |  |
| 9 | Shigella ~ HE | 51.77 | 51.9 | 6.38 | 0.01 | 98 | HE | 3.17 | 1.88 | 0.09 |  |
| **10** | **Shigella ~ ADO** | **47.30** | **47.43** | **1.91** | **0.06** | **98** | **ADO** | **4.53** | **1.72** | **0.01**** |  |
| 11 | Shigella ~ ADI | 53.44 | 53.56 | 8.04 | 0.00 | 98 | ADI | -2.35 | 2.13 | 0.27 |  |
| **12** | **Shigella ~ ASO** | **46.53** | **46.65** | **1.13** | **0.09** | **98** | **ASO** | **4.56** | **1.61** | **0.01**** |  |
| 13 | Shigella ~ ASI | 54.12 | 54.24 | 8.72 | 0.00 | 98 | ASI | -1.91 | 2.55 | 0.45 |  |
| 14 | Shigella ~ DC * GO | 53.30 | 53.72 | 8.2 | 0.00 | 96 | DC | 6.17 | 4.57 | 0.18 |  |
|  |  |  |  |  |  |  | GO | 2.31 | 3.72 | 0.54 |  |
|  |  |  |  |  |  |  | DC*GO | -4.05 | 7.16 | 0.57 |  |
| 15 | Shigella ~ DC * GI | 52.71 | 53.13 | 7.61 | 0.00 | 96 | DC | 1.35 | 3.97 | 0.73 |  |
|  |  |  |  |  |  |  | GI | 0.11 | 3.52 | 0.97 |  |
|  |  |  |  |  |  |  | DC*GI | 3.53 | 6.91 | 0.61 |  |
| 16 | Shigella ~ DC * GB | 52.34 | 53.76 | 8.24 | 0.00 | 96 | DC | 4.20 | 3.72 | 0.26 |  |
|  |  |  |  |  |  |  | GB | 1.99 | 4.03 | 0.62 |  |
|  |  |  |  |  |  |  | DC*GB | -1.99 | 10.22 | 0.85 |  |
| 17 | Shigella ~ DC * GE | 53.00 | 53.42 | 7.9 | 0.00 | 96 | DC | 1.40 | 4.51 | 0.76 |  |
|  |  |  |  |  |  |  | GE | -0.10 | 3.21 | 0.97 |  |
|  |  |  |  |  |  |  | DC*GE | 2.94 | 6.44 | 0.65 |  |
| 18 | Shigella ~ DC * HD | 49.94 | 50.36 | 4.84 | 0.01 | 96 | DC | -4.45 | 6.96 | 0.52 |  |
|  |  |  |  |  |  |  | HD | -1.15 | 4.00 | 0.77 |  |
|  |  |  |  |  |  |  | DC*HD | 13.64 | 11.69 | 0.24 |  |
| 19 | Shigella ~ DC * HB | 49.34 | 49.76 | 4.24 | 0.02 | 96 | DC | 0.70 | 4.27 | 0.87 |  |
|  |  |  |  |  |  |  | HB | 0.25 | 3.82 | 0.95 |  |
|  |  |  |  |  |  |  | DC*HB | 10.43 | 10.93 | 0.34 |  |
| 20 | Shigella ~ DC * HE | 51.35 | 51.77 | 6.25 | 0.01 | 96 | DC | -2.59 | 6.66 | 0.70 |  |
|  |  |  |  |  |  |  | HE | -0.73 | 3.98 | 0.86 |  |
|  |  |  |  |  |  |  | DC*HE | 9.84 | 10.96 | 0.37 |  |
| 21 | Shigella ~ GO + ADO | 49.03 | 49.28 | 3.76 | 0.02 | 97 | GO | 1.09 | 2.03 | 0.59 |  |
|  |  |  |  |  |  |  | ADO | 4.11 | 1.87 | 0.03* |  |
| 22 | Shigella ~ GO + ADI | 53.93 | 54.18 | 8.66 | 0.00 | 97 | GO | 2.32 | 1.89 | 0.22 |  |
|  |  |  |  |  |  |  | ADI | -1.07 | 2.40 | 0.66 |  |
| 23 | Shigella ~ GO + ASO | 48.28 | 48.53 | 3.01 | 0.04 | 97 | GO | 1.05 | 2.09 | 0.62 |  |
|  |  |  |  |  |  |  | ASO | 4.16 | 1.77 | 0.02* |  |
| 24 | Shigella ~ GO + ASI | 54.05 | 54.30 | 8.78 | 0.00 | 97 | GO | 2.56 | 1.76 | 0.15 |  |
|  |  |  |  |  |  |  | ASI | -0.71 | 2.56 | 0.78 |  |
| 25 | Shigella ~ GI + ADO | 49.19 | 49.44 | 3.92 | 0.02 | 97 | GI | -0.68 | 2.03 | 0.74 |  |
|  |  |  |  |  |  |  | ADO | 4.73 | 1.83 | 0.01** |  |
| 26 | Shigella ~ GI + ADI | 55.32 | 55.57 | 10.05 | 0.00 | 97 | GI | 0.60 | 1.77 | 0.73 |  |
|  |  |  |  |  |  |  | ADI | -2.26 | 2.15 | 0.29 |  |
| 27 | Shigella ~ GI + ASO | 48.42 | 48.67 | 3.15 | 0.03 | 97 | GI | -0.67 | 2.06 | 0.74 |  |
|  |  |  |  |  |  |  | ASO | 4.74 | 1.70 | 0.01** |  |
| 28 | Shigella ~ GI + ASI | 55.91 | 56.16 | 10.64 | 0.00 | 97 | GI | 0.79 | 1.74 | 0.65 |  |
|  |  |  |  |  |  |  | ASI | -1.86 | 2.56 | 0.47 |  |
| 29 | Shigella ~ GB + ADO | 49.18 | 49.43 | 3.91 | 0.02 | 97 | GB | 0.85 | 2.34 | 0.72 |  |
|  |  |  |  |  |  |  | ADO | 4.37 | 1.77 | 0.01** |  |
| 30 | Shigella ~ GB + ADI | 54.91 | 55.16 | 9.64 | 0.00 | 97 | GB | 1.50 | 1.97 | 0.45 |  |
|  |  |  |  |  |  |  | ADI | -1.93 | 2.22 | 0.38 |  |
| 31 | Shigella ~ GB + ASO | 48.40 | 48.65 | 3.14 | 0.03 | 97 | GB | 0.88 | 2.44 | 0.72 |  |
|  |  |  |  |  |  |  | ASO | 4.40 | 1.67 | 0.01** |  |
| 32 | Shigella ~ GB + ASI | 55.31 | 55.56 | 10.04 | 0.00 | 97 | GB | 1.80 | 1.89 | 0.34 |  |
|  |  |  |  |  |  |  | ASI | -1.55 | 2.57 | 0.55 |  |
| 33 | Shigella ~ GE + ADO | 49.29 | 49.54 | 4.02 | 0.02 | 97 | GE | 0.26 | 1.98 | 0.90 |  |
|  |  |  |  |  |  |  | ADO | 4.40 | 2.01 | 0.03* |  |
| 34 | Shigella ~ GE + ADI | 54.30 | 54.55 | 9.03 | 0.00 | 97 | GE | 1.85 | 1.76 | 0.29 |  |
|  |  |  |  |  |  |  | ADI | -1.19 | 2.43 | 0.62 |  |
| 35 | Shigella ~ GE + ASO | 48.51 | 48.76 | 3.25 | 0.03 | 97 | GE | 0.21 | 2.03 | 0.92 |  |
|  |  |  |  |  |  |  | ASO | 4.45 | 1.91 | 0.02* |  |
| 36 | Shigella ~ GE + ASI | 54.44 | 54.69 | 9.17 | 0.00 | 97 | GE | 2.10 | 1.65 | 0.20 |  |
|  |  |  |  |  |  |  | ASI | -0.82 | 2.62 | 0.75 |  |
| 37 | Shigella ~ HD + ADO | 47.29 | 47.54 | 2.03 | 0.06 | 97 | HD | 3.01 | 2.14 | 0.16 |  |
|  |  |  |  |  |  |  | ADO | 4.25 | 1.77 | 0.02* |  |
| 38 | Shigella ~ HD + ADI | 53.13 | 53.38 | 7.86 | 0.00 | 97 | HD | 2.94 | 1.99 | 0.14 |  |
|  |  |  |  |  |  |  | ADI | -1.25 | 2.36 | 0.60 |  |
| 39 | Shigella ~ HD + ASO | 46.55 | 46.80 | 1.28 | 0.08 | 97 | HD | 3.04 | 2.19 | 0.16 |  |
|  |  |  |  |  |  |  | ASO | 4.25 | 1.65 | 0.01** |  |
| 40 | Shigella ~ HD + ASI | 53.34 | 53.59 | 8.07 | 0.00 | 97 | HD | 3.15 | 1.96 | 0.11 |  |
|  |  |  |  |  |  |  | ASI | -0.75 | 2.81 | 0.79 |  |
| **41** | **Shigella ~ HB + ADO** | **46.17** | **46.42** | **0.90** | **0.10** | **97** | **HB** | **3.31** | **1.85** | **0.07** |  |
|  |  |  |  |  |  |  | **ADO** | **5.12** | **1.95** | **0.01**** |  |
| 42 | Shigella ~ HB + ADI | 53.44 | 53.69 | 8.17 | 0.00 | 97 | HB | 2.33 | 1.60 | 0.15 |  |
|  |  |  |  |  |  |  | ADI | -1.93 | 2.28 | 0.40 |  |
| **43** | **Shigella ~ HB + ASO** | **45.27** | **45.52** | **0.00** | **0.16** | **97** | **HB** | **3.43** | **1.87** | **0.07** |  |
|  |  |  |  |  |  |  | **ASO** | **5.09** | **1.81** | **0.00**** |  |
| 44 | Shigella ~ HB + ASI | 53.87 | 54.12 | 8.60 | 0.00 | 97 | HB | 2.43 | 1.57 | 0.12 |  |
|  |  |  |  |  |  |  | ASI | -1.51 | 2.75 | 0.58 |  |
| 45 | Shigella ~ HE + ADO | 53.87 | 54.12 | 8.60 | 0.00 | 97 | HE | 2.31 | 2.14 | 0.28 |  |
|  |  |  |  |  |  |  | ADO | 4.07 | 1.77 | 0.02* |  |
| 46 | Shigella ~ HE + ADI | 53.57 | 53.82 | 8.30 | 0.00 | 97 | HE | 2.76 | 2.07 | 0.18 |  |
|  |  |  |  |  |  |  | ADI | -1.08 | 2.40 | 0.65 |  |
| 47 | Shigella ~ HE + ASO | 47.43 | 47.68 | 2.16 | 0.05 | 97 | HE | 2.29 | 2.18 | 0.29 |  |
|  |  |  |  |  |  |  | ASO | 4.09 | 1.66 | 0.01** |  |
| 48 | Shigella ~ HE + ASI | 53.73 | 53.98 | 8.47 | 0.00 | 97 | HE | 3.02 | 2.02 | 0.14 |  |
|  |  |  |  |  |  |  | ASI | -0.54 | 2.80 | 0.85 |  |

DR: Dominance Rank; DC: Dominance Certainty; GO: Groom Outdegree; GI: Groom Indegree; GB: Groom Betweenness; GE: Groom Eigenvector; HD: Huddle Degree; HB: Huddle Betweenness; HE: Huddle Eigenvector; ADO: Aggression Outdegree; ADI: Aggression Indegree; ASO: Aggression Outstrength; ASI: Aggression Instrength

Δ: Difference in AICc from the best-fit model

W: Absolute model weight

*p < 0.05 **p < 0.01
